# FMRP-dependent production of large dosage-sensitive proteins is highly conserved

**DOI:** 10.1093/genetics/iyac094

**Published:** 2022-06-22

**Authors:** Keegan Flanagan, Alireza Baradaran-Heravi, Qi Yin, Khanh Dao Duc, Allan C Spradling, Ethan J Greenblatt

**Affiliations:** Department of Biochemistry and Molecular Biology, University of British Columbia, Vancouver, BC V6T 1Z3, Canada; Department of Mathematics, University of British Columbia, Vancouver, BC V6T 1Z2, Canada; Department of Biochemistry and Molecular Biology, University of British Columbia, Vancouver, BC V6T 1Z3, Canada; Howard Hughes Medical Institute Research Laboratories, Department of Embryology, Carnegie Institution for Science, Baltimore, MD 21218 USA; Department of Mathematics, University of British Columbia, Vancouver, BC V6T 1Z2, Canada; Howard Hughes Medical Institute Research Laboratories, Department of Embryology, Carnegie Institution for Science, Baltimore, MD 21218 USA; Department of Biochemistry and Molecular Biology, University of British Columbia, Vancouver, BC V6T 1Z3, Canada; Howard Hughes Medical Institute Research Laboratories, Department of Embryology, Carnegie Institution for Science, Baltimore, MD 21218 USA

**Keywords:** Fmr1, FMRP, autism, RNP, translation

## Abstract

Mutations in *FMR1* are the most common heritable cause of autism spectrum disorder. *FMR1* encodes an RNA-binding protein, FMRP, which binds to long, autism-relevant transcripts and is essential for normal neuronal and ovarian development. In contrast to the prevailing model that FMRP acts to block translation elongation, we previously found that FMRP activates the translation initiation of large proteins in *Drosophila* oocytes. We now provide evidence that FMRP-dependent translation is conserved and occurs in the mammalian brain. Our comparisons of the mammalian cortex and *Drosophila* oocyte ribosome profiling data show that translation of FMRP-bound mRNAs decreases to a similar magnitude in FMRP-deficient tissues from both species. The steady-state levels of several FMRP targets were reduced in the *Fmr1* KO mouse cortex, including a ∼50% reduction of Auts2, a gene implicated in an autosomal dominant autism spectrum disorder. To distinguish between effects on elongation and initiation, we used a novel metric to detect the rate-limiting ribosome stalling. We found no evidence that FMRP target protein production is governed by translation elongation rates. FMRP translational activation of large proteins may be critical for normal human development, as more than 20 FMRP targets including Auts2 are dosage sensitive and are associated with neurodevelopmental disorders caused by haploinsufficiency.

## Teaser

Mutations in FMR1, which encodes RNA binding protein FMRP, lead to fragile X syndrome: the most common cause of autism spectrum disorder. Flanagan, Baradaran-Hervari *et al*. previously found that FMRP promotes translation initiation of large proteins in *Drosophila* oocytes—in contrast to its previously ascribed function as a repressor of translation elongation. By comparing ribosome profiling data and measuring steady state levels of FMRP targets, they now show that FMRP-dependent large protein translation is highly conserved in the mammalian cortex.

## Introduction

Mutations in the *FMR1* gene, encoding the RNA-binding protein FMRP, lead to fragile X syndrome (FXS) and fragile X primary ovarian insufficiency, causes of intellectual disability (ID), autism spectrum disorder (ASD), and premature ovarian failure (Sullivan *et al.* 2011; Hagerman *et al.* 2017). FMRP binds to hundreds of individual mRNAs, which are longer than average and which are nonrandomly enriched for genes associated with ID and ASD (Darnell *et al.* 2011; Ascano *et al.* 2012). FMRP loss leads to a wide array of phenotypes in *Fmr1* knockout animals, which extend beyond changes in translation. These include altered RNA transport (Kao *et al.* 2010; Goering *et al.* 2020), RNA nuclear export(Hsu *et al.* 2019), RNA stability (Shu *et al.* 2020), metabolic signaling (Sharma *et al.* 2010), ion channel function (Brager *et al.* 2012), stem cell proliferation (Callan *et al.* 2010), dendritic spine maturation (Grossman *et al.* 2006), homeostatic plasticity (Zhang *et al.* 2018; Bülow *et al.* 2019), and mGluR5-dependent long-term depression (Osterweil *et al.* 2010; Hays *et al.* 2011; Prilutsky *et al.* 2015). Treatments targeting the primary defects associated with FMRP loss have the potential to alleviate secondary effects as well. However, it has been difficult to determine which phenotypes are a primary result of *Fmr1* loss and are a secondary result of the dysregulation of 1 or more of hundreds of potential FMRP target genes. FMRP’s precise molecular function and key targets have remained controversial (Aryal and Klann 2018).

FMRP associates with polysomes, suggesting a role for FMRP in translational regulation. *In vitro* measurements of translation of neuronal mRNAs performed in reticulocyte extracts showed widespread changes in translation elongation rates in the absence of FMRP (Darnell *et al.* 2011); however, experiments determining translation elongation rates are performed in the presence of translation initiation inhibitors, confounding their interpretation. Measurements of *Drosophila* oocyte protein production with ribosome profiling revealed that FMRP preferentially affects large proteins and that it promotes rather than represses translation by promoting the translation initiation (Greenblatt and Spradling 2018). These divergent effects of FMRP in mice and *Drosophila* could be due to species and/or tissue differences. However, FMRP is highly conserved in structure and function between *Drosophila*, mouse, and many other species, and the human *FMR1* gene rescues neuronal defects in *Drosophila Fmr1* mutants (Coffee *et al.* 2010). Moreover, long protein-coding capacity is emerging as a common property shared by most mRNA targets regulated by FMRP in multiple systems (Greenblatt and Spradling 2018; Das Sharma *et al.* 2019; Sears *et al.* 2019; Aryal *et al.* 2021).

By reanalyzing ribosome profiling data using a common pipeline from experiments conducted with FMRP-deficient *Drosophila* oocytes (Greenblatt and Spradling 2018) or mouse cortex (Das Sharma *et al.* 2019), we find that ribosome footprints of FMRP targets encoding large proteins are reduced by comparable magnitudes, suggesting a conserved role of FMRP in translational activation. Consistent with an activator function, large FMRP target proteins examined using capillary electrophoresis immunoassays showed reduced steady-state levels in the cortex of *Fmr1* KO mice. These targets include Auts2, which is decreased by ∼50% and which is associated with a human autism disorder caused by *Auts2* haploinsufficiency.

The decrease in translation initiation rates of FMRP targets contrasts with previous models suggesting that FMRP acts as a repressor of translation elongation. To determine whether translation of FMRP targets is limited by translation initiation or elongation rates, we developed a bioinformatic method to identify changes in ribosome distribution that would be consistent with the alleviation of rate-limiting stalling in FMRP-deficient tissues. In contrast to the elongation stalling model and consistent with the translation initiation of FMRP targets being rate limiting, we find that the distribution of ribosomes along FMRP target transcripts is nearly identical in control and FMRP-deficient tissues.

Finally, we find that ASD-relevant genes generally encode proteins that are larger than average and that many FMRP ASD target genes are haploinsufficient but not triplosensitive. Our findings are consistent with an ancient and conserved role for FMRP to promote the production of large, dosage-sensitive proteins essential for normal neuronal development. Our data support a model in which FMRP's primary function is to increase the rate of translation initiation of its targets.

## Methods

### Read alignments and quantification

Raw sequencing data (Woolstenhulme *et al.* 2015; Fradejas-Villar *et al.* 2017; Greenblatt and Spradling 2018; Das Sharma *et al.* 2019; Philippe *et al.* 2020) from fly, mouse, *Escherichiacoli*, and human samples were aligned to the Flybase Consortium/Berkeley *Drosophila* Genome Project (BDGP)/Celera Genomics *Drosophila* release dm 6.28, Genome Reference Consortium mouse build 38 mm10, University of Wisconsin *E.coli* strain K-12 sub-strain MG1655 build ASM584v2, and Genome Reference Consortium Human build 38 assemblies, respectively. Alignments were performed with STAR (Dobin *et al.* 2013). The aligned data from both the mRNA sequencing and ribosome footprinting experiments were imported into Python and quantified using the Plastid (Dunn and Weissman 2016) software package.

### Length and expression analyses

SFARI Class I and Class II genes were obtained from the SFARI Gene database (Abrahams *et al.* 2013). For each gene, the highest expressed transcript isoform was used as the representative form. Transcripts with a transcript per million (TPM) value of <2 were excluded from analyses. Intron, untranslated region (UTR), and coding sequence (CDS) lengths for transcript isoforms were computed from BDGP and refGene gene models for *Drosophila* and mouse data, respectively. For translation efficiency analyses, fold-change and *P*_adj_ values were computed using RiboDiff software (Zhong *et al.* 2017). Stress-granule enrichment data were obtained from Khong *et al.* (2017).

### Ribosome stalling analysis

Ribosome footprints were mapped to their P-sites with 3′ offsets that varied based on the length of the individual ribosome footprints (Supplementary Table 5). These variable 3′ offsets were determined using the psite function from the riboWaltz R package (Lauria *et al.* 2018). Ribosome footprinting read densities were determined using the Plastid software package. These densities were then smoothed using LOWESS regression and normalized by the total number of reads per transcript. Transcripts were filtered so that only transcripts with a length exceeding 100 codons and an average read density above 0.5 reads/codon were kept. The Kolmogorov–Smirnov (K–S) statistic for all transcripts was calculated as the maximum absolute difference between the control and mutant normalized read densities. All transcripts were grouped into high (>0.3), medium (>0.3 and <0.15), and low (<0.15) K–S statistic groups. The fold enrichment of targets in each group was determined as the fraction of the total targets in that group over the fraction of the total nontargets in that group. The significance of target enrichment was determined using Fisher’s exact test.

### Simulations with the inhomogeneous ℓ-Totally Asymmetric Simple Exclusion Process model

Plausible elongation rates for each codon position along simulated transcripts were computed from a gamma distribution to mimic the observed distributions of elongation rates. Elongation rates of control transcripts were greatly reduced at random to simulate a mutation inducing pauses in translation. These elongation rates were inputted into an implementation of the inhomogeneous ℓ-Totally Asymmetric Simple Exclusion Process (TASEP) (Erdmann-Pham *et al.* 2020; Erdmann-Pham *et al.* 2021) model in Python. The inhomogeneous ℓ-TASEP model was used to determine if translation was initiation limited, elongation limited, or termination limited. Simulations using the inhomogeneous ℓ-TASEP model were performed to obtain ribosome densities across the simulated transcript. Finally, the transcript was undersampled to simulate the process of obtaining a ribosome footprint. This simulation was repeated many times to obtain many different simulated ribosome footprints for transcripts of varying lengths.

### Mouse brain cortex dissection and capillary electrophoresis western experiments

The animal experiments were carried out in accordance with the protocols of Carnegie Institution of Washington (CIW) IACUC. All mice were kept in specific pathogen-free facilities of CIW. *Fmr1* knockout mice (003025) were acquired from the Jackson Laboratory. For brain cortex dissection, 24-day-old male mice were scarified using cervical dislocation. The brains were collected after breaking off the skull and the meninges, followed by freeing the cortical hemisphere from the brain through removing the cerebellum and the pons. The cortex was then dissected, frozen in liquid nitrogen, and stored in −80°C. Samples were lysed in 0.5 ml of cell extraction buffer (FNN0011; Thermo Fisher Scientific) supplemented with 1× complete protease inhibitor cocktail (Roche Molecular Biochemicals) with a Dounce homogenizer on ice. After 30-min incubation on ice, with vortexing at 10-min intervals, lysate was centrifuged at 13,000 rpm for 10 min at 4°C and the supernatant was collected for total protein quantification. The western analysis assays for FMRP, Auts2, Med13L, HUWE1, Arid2, Arid1a, Fat1, Ubr4, and Sptbn2 detection were performed as previously described (Baradaran-Heravi *et al.* 2016) with minor modifications. In brief, mixtures of cell lysates (0.5 or 1 mg/ml, diluted in 0.1× WES sample buffer) and the fluorescent master mix were heated at 70°C for 5 min. The samples, blocking and chemiluminescent reagents, primary and secondary antibodies, and wash buffer were dispensed into the microplates with 12–230 or 66–440 kDa separation modules and capillary electrophoresis western analysis was carried out with the ProteinSimple WES instrument. Total protein in the lysates was also measured similarly followed by biotin labeling of all proteins. Rabbit anti-FMRP (1:50; Cell Signaling 4317), rabbit anti-Auts2 (1:50; Abcam ab96326), rabbit anti-Med13L (1:50; Bethyl Laboratories A302-420A), rabbit anti-HUWE1 (1:100; Thermo Fisher Scientific A300-486A), rabbit anti-Arid2 (1:50; Abiocode R2380-1), mouse anti-Arid1a (1:50; Santa Cruz sc-32761), rabbit anti-Fat1 (1:50; Abcam ab241372), rabbit anti-Ubr4 antibody (1:100; Abcam ab86738), and rabbit anti-Sptbn2 (1:50; Proteinteck 55107-1-AP) antibodies were used. The data were acquired in electropherograms and analyzed using the inbuilt Compass software (ProteinSimple). The expression level of each target protein was first adjusted using the concentration curve expression generated for 1 wild-type sample and then normalized using the total biotin-labeled protein detected in the same volume of each lysate.

## Results

### The effect of FMRP deficiency on translation is strikingly similar in *Drosophila* oocytes and the mouse cortex

**Fig. 1.**
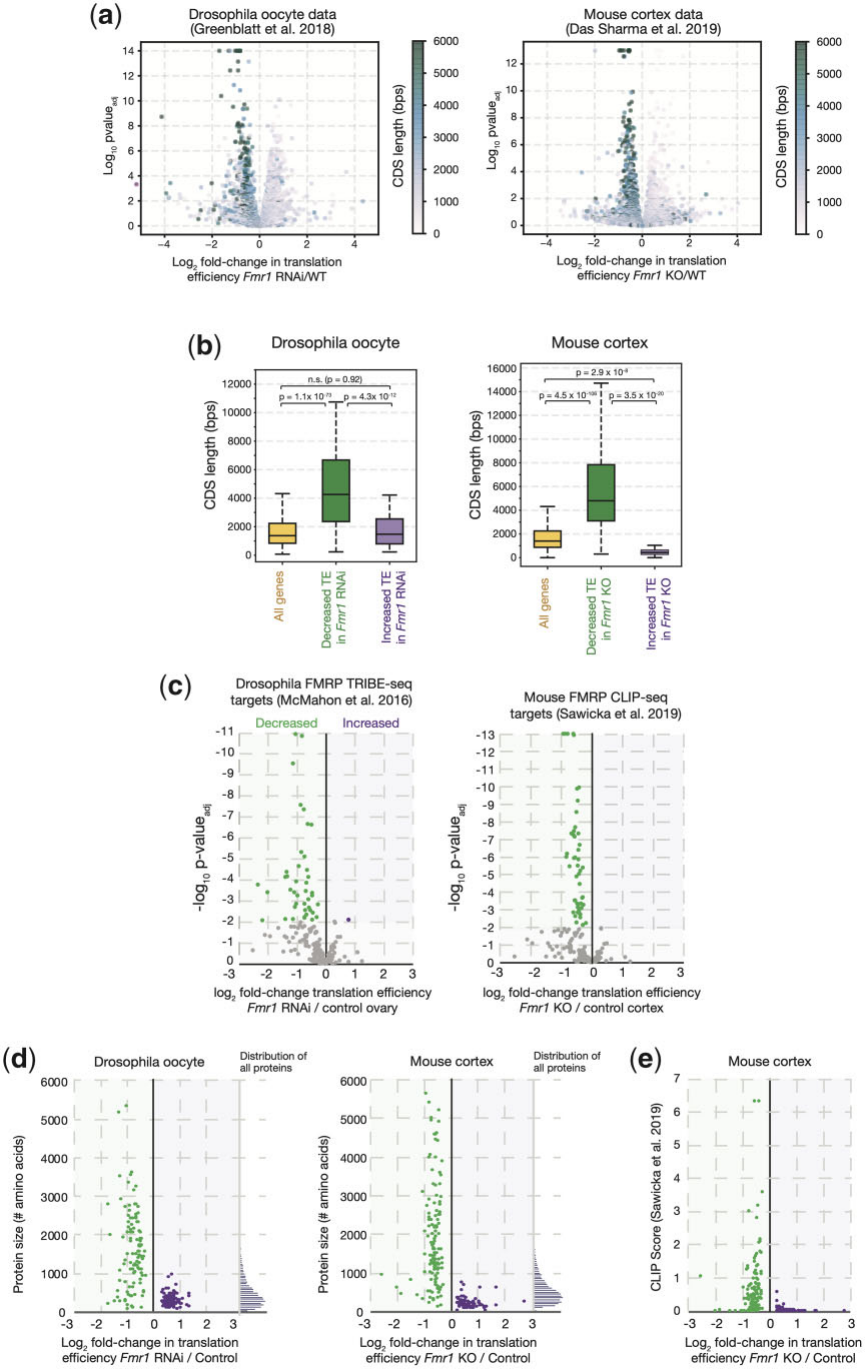
FMRP has a highly conserved role to promote translation in *Drosophila* oocytes and the mouse cortex. a) Volcano and b) box plots showing concordantly diminished translation efficiency of mRNAs with long CDS regions in FMRP-deficient *Drosophila* oocytes (Greenblatt and Spradling 2018) and mouse cortex (Das Sharma *et al.* 2019). c) Volcano plots showing concordantly diminished translation efficiency of FMRP-bound mRNAs (McMahon *et al.* 2016; Liu *et al.* 2018) in FMRP-deficient *Drosophila* oocytes and in mouse cortex. d) Scatter plots showing reduced translation efficiency as a function of protein size for the 200 most significantly affected genes in *Fmr1* RNAi *Drosophila* oocytes or *FMR1* KO mouse cortex. Histograms of size distributions for all *Drosophila* or mouse proteins respectively are shown to the right. e) Scatter plot showing reduced translation efficiency as a function of CLIP score (Sawicka *et al.* 2019) for the 200 most significantly affected genes in *the FMR1* KO mouse cortex.

To test whether FMRP has a similar function in *Drosophila* oocytes and in mouse cortex, we compared the effects of FMRP on translation as measured using ribosome profiling in these 2 tissues and organisms (Greenblatt and Spradling 2018; Das Sharma *et al.* 2019) but re-analyzed here from primary reads with identical bioinformatic pipelines (see *Methods* and Supplementary Table 1). In this analysis, the translation efficiency is considered to be proportional to the frequency of ribosome footprints mapped onto the mRNA in question by ribosome profiling normalized to mRNA levels as measured by mRNA-seq. Consistent with Greenblatt *et al.*, we found that many transcripts containing long CDS were translationally downregulated in *Fmr1* RNAi oocytes as compared to controls (Fig. 1a). The median CDS length of genes whose translation decreased significantly in *Fmr1* RNAi oocytes was 3.1 times longer than the median length of all oocyte expressed genes (4,262 vs 1,372 bp, respectively, Fig. 1b). We observed a strikingly similar effect of *Fmr1* KO in the mouse cortex. Many genes encoding large proteins were translationally downregulated over a similar range (Fig. 1a), and the affected mRNAs had 3.4 times longer CDS lengths as compared to all cortex-expressed genes (4,797 vs 1,401 bp, respectively, Fig. 1b).

If FMRP acts directly to activate translation, then mRNAs bound by FMRP should be selectively reduced rather than increased in translation in mutant tissues. Consistent with FMRP acting directly, mouse cortex mRNAs bound by FMRP had reduced ribosome footprints in *Fmr1*-null animals (Das Sharma *et al.* 2019). Likewise, we found that *Drosophila* and mouse FMRP direct targets, as determined by TRIBE (McMahon *et al.* 2016) and in more recent CLIP-seq experiments (Sawicka *et al.* 2019), were concordantly translationally downregulated. Ninety-eight percentage (43/44) and 100% (50/50) of translationally altered mRNAs bound by FMRP in *Drosophila* oocytes and the mouse hippocampus, respectively, exhibited reduced translation efficiency in the absence of FMRP (Fig. 1c). FMRP CLIP targets were more than 10 times more likely to be translationally reduced in the *Fmr1* mutant mouse cortex compared to all neuronally expressed transcripts irrespective of FMRP binding (25% vs 2.2% respectively, *P* = 3.8 × 10^−107^, χ^2^ test). These data argue that FMRP acts directly to increase the translation of a subset of bound mRNAs.

### The magnitude of FMRP translational activation is similar for targets of varying length

FMRP contains multiple RNA-binding domains and studies suggest that it has the potential to bind to widespread tetranucleotide motifs and G-rich sequences (Ascano *et al.* 2012; Vasilyev *et al.* 2015; Anderson *et al.* 2016). FMRP-dependent translational activation might depend on length, with longer mRNAs being more strongly affected on average simply due to a greater number of FMRP-binding sites. An alternative possibility is that FMRP translational activation requires only that a minimum number of binding sites be occupied by FMRP, reflecting a highly cooperative process. This latter model predicts that FMRP translational activation would be independent of mRNA length, at least beyond a threshold (i.e. the length at which the minimum number of binding sites is occupied). To distinguish between these possibilities, we compared the magnitude of downregulation of translation for transcripts of varying length in FMRP-deficient cells. We found that while genes encoding large proteins were more likely to be FMRP targets, the magnitude of the reduction in translation did not increase proportionately with CDS length (Fig. 1d). The magnitude of reduction in translation was similar for FMRP targets of varying length (Fig. 1d) or CLIP score (Fig. 1e), an indicator of the strength of FMRP binding. These data support a threshold model for FMRP-dependent translational activation in both *Drosophila* oocytes and the mouse cortex.

### FMRP targets show decreased expression in the mouse cortex

**Fig. 2.**
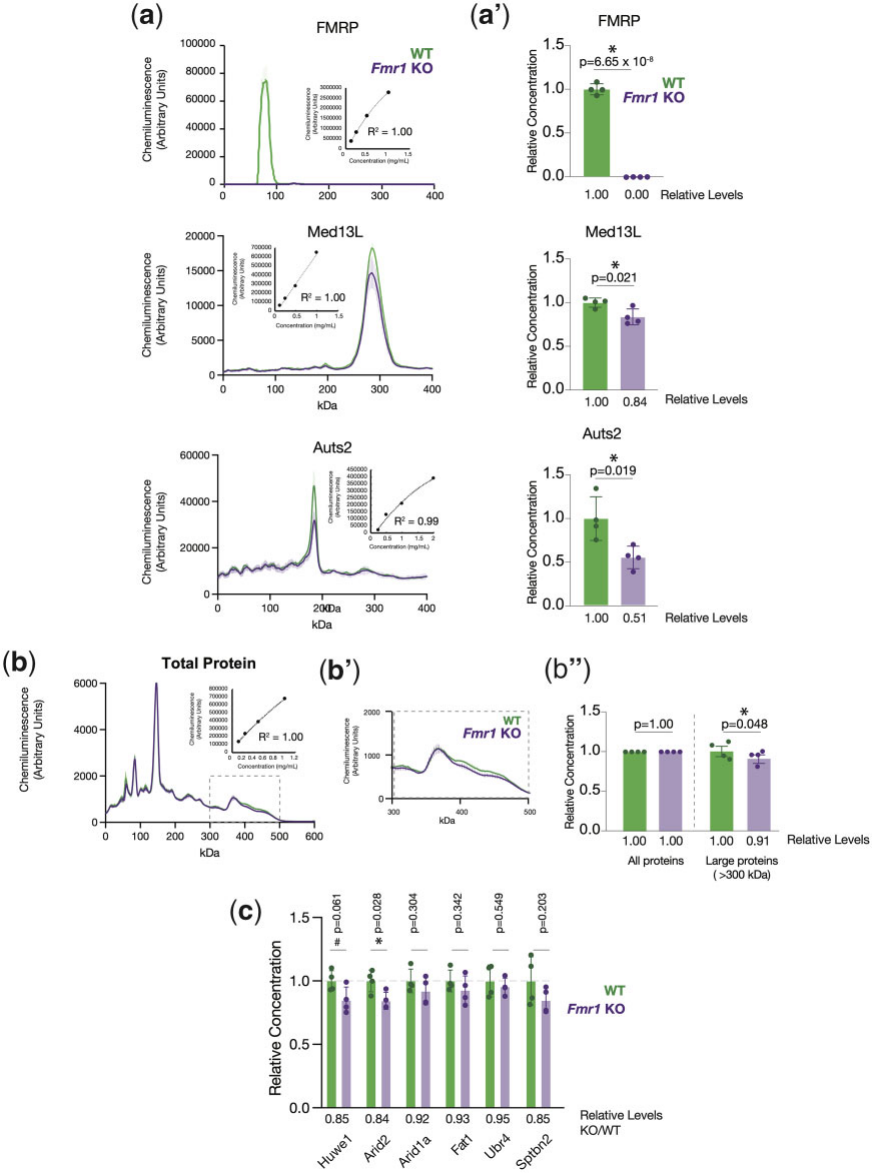
FMRP targets encoding large proteins are downregulated in the mouse cortex. a, a′) Profiles and quantification of capillary electrophoresis western experiments showing a partial reduction in the levels of FMRP targets Auts2 and Med13L and complete loss of FMRP in the *Fmr1* KO mouse cortex as compared to wild-type controls. Insets show standard curves of western signal as a function of total protein concentration. b) Total protein levels of all biotin-labeled proteins in mouse cortex extracts. b′) A zoom-in of (b) showing large proteins >300 kDa. b″) Quantification of (b) showing a decrease in the levels of large proteins but not total proteins in *Fmr1* KO mouse cortex samples. c) Quantification of capillary electrophoresis western experiments of indicated proteins performed on mouse cortex extracts from wild-type or *Fmr1* KO animals. *P < 0.05, ^#^*P* < 0.1.

To further test whether FMRP acts to promote the translation of large proteins in the mouse cortex, we performed western experiments to determine the steady-state levels of FMRP direct targets as determined by CLIP-seq (Darnell *et al.* 2011; Li *et al.* 2020) in wild-type or FMRP-deficient tissues. We used automated capillary electrophoresis western analysis, which allows for the efficient detection of large proteins as it avoids a potentially lossy electrophoretic transfer step. The steady-state levels of all FMRP targets tested were reduced in FMRP KO cortex samples between 5% and 50% (Fig. 2, a–c). The largest reduction (49% reduced, *P* = 0.019, *t*-test) was observed for Auts2 (Fig. 2, a and a′). *Auts2* encodes a PRC1-associated transcriptional activator essential for normal neuronal gene expression (Gao *et al.* 2014), and haploinsufficiency of *Auts2* is associated with ID and autism (Oksenberg and Ahituv 2013). The protein levels of Med13L, HUWE1, and Arid2 were also reduced by 15–17% in the FMRP KO cortex as compared to controls (Fig. 2, a–c) (*P* = 0.021, *P* = 0.061, and *P* = 0.028 respectively). *Med13L* encodes a subunit of the Mediator complex required for neural crest cell migration (Utami *et al.* 2014), *Arid2* is a core subunit of the PBAF chromatin remodeling complex whose loss results in neuronal hyperconnectivity (Zaslavsky *et al.* 2019), and *HUWE1* encodes a large ubiquitin ligase, which inhibits neuronal progenitor proliferation (Zhao *et al.* 2008). All 3 of these genes are implicated in ID and autism disorders, and *Med13L* and *Arid2* mutations are associated with dominant disorders caused by *Med13L*/*Arid2* haploinsufficiency (Utami *et al.* 2014; Bramswig *et al.* 2017). Consistent with FMRP promoting the translation of a fraction of large proteins in the brain, we observed a small reduction (9%) in the bulk levels of all cellular proteins >300 kDa labeled with biotin (Fig. 2, b′ and b″). The levels of other FMRP CLIP targets, *Fat1*, *Ubr4*, and *Sptbn2* also exhibited a trend toward reduced rather than increased levels (Fig. 2c). No FMRP target among the 8 tested had increased cortex protein levels in the absence of FMRP. Together, these data indicate that multiple FMRP targets encoding dosage-sensitive genes essential for neurodevelopment are downregulated in the FMRP KO mouse brain.

### A novel method to detect changes in translation due to ribosomal stalling

Declines in ribosome footprints on target mRNAs in FMRP-deficient tissues could in theory result from reduced translation initiation or increased elongation relative to controls. Similar to our measurements performed in the mouse cortex (Fig. 2, a–c), *Drosophila* oocytes and neurons are known to contain lower levels of 3 large FMRP target proteins in FMRP-deficient cells, consistent with reduced translation initiation (Greenblatt and Spradling 2018; Sears *et al.* 2019). However, ribosome density was reported to be more uniform across translated mRNAs in FMRP-deficient neurons (Das Sharma *et al.* 2019), a finding potentially consistent with a role for FMRP in translation elongation. Translation elongation rates are typically measured in the presence of inhibitors of translation initiation, confounding the interpretation of these experiments (Schmidt *et al.* 2009; Darnell *et al.* 2011). Observed changes in translation elongation may not impact overall protein production rates if translation initiation rather than elongation is rate-limiting. While translation of the vast majority of mRNAs is initiation limited (Ingolia *et al.* 2011; Shah *et al.* 2013; Erdmann-Pham *et al.* 2020), elongation limitation has been shown to occur in cells with low levels of tRNAs (Darnell *et al.* 2018) or in the absence of elongation factors (Woolstenhulme *et al.* 2015; Schuller *et al.* 2017).

To disentangle the effects of differential ribosome pausing and reduced translation initiation in FMRP-lacking cells, we sought to develop a quantitative method to detect rate-limiting pause sites using published ribosome profiling data. Rate-limiting pausing during translation elongation leads to the accumulation of stacked ribosomes prior to the pause site (Wolin and Walter 1988). Similar to the distribution of cars before or after a traffic jam, high densities of ribosomes precede rate-limiting pause sites and fewer ribosomes are found downstream of the pause site (Woolstenhulme *et al.* 2015; Erdmann-Pham *et al.* 2020). The appearance of “ribosome traffic jams” is therefore indicative of the existence of a rate-limiting ribosomal pause site.

**Fig. 3.**
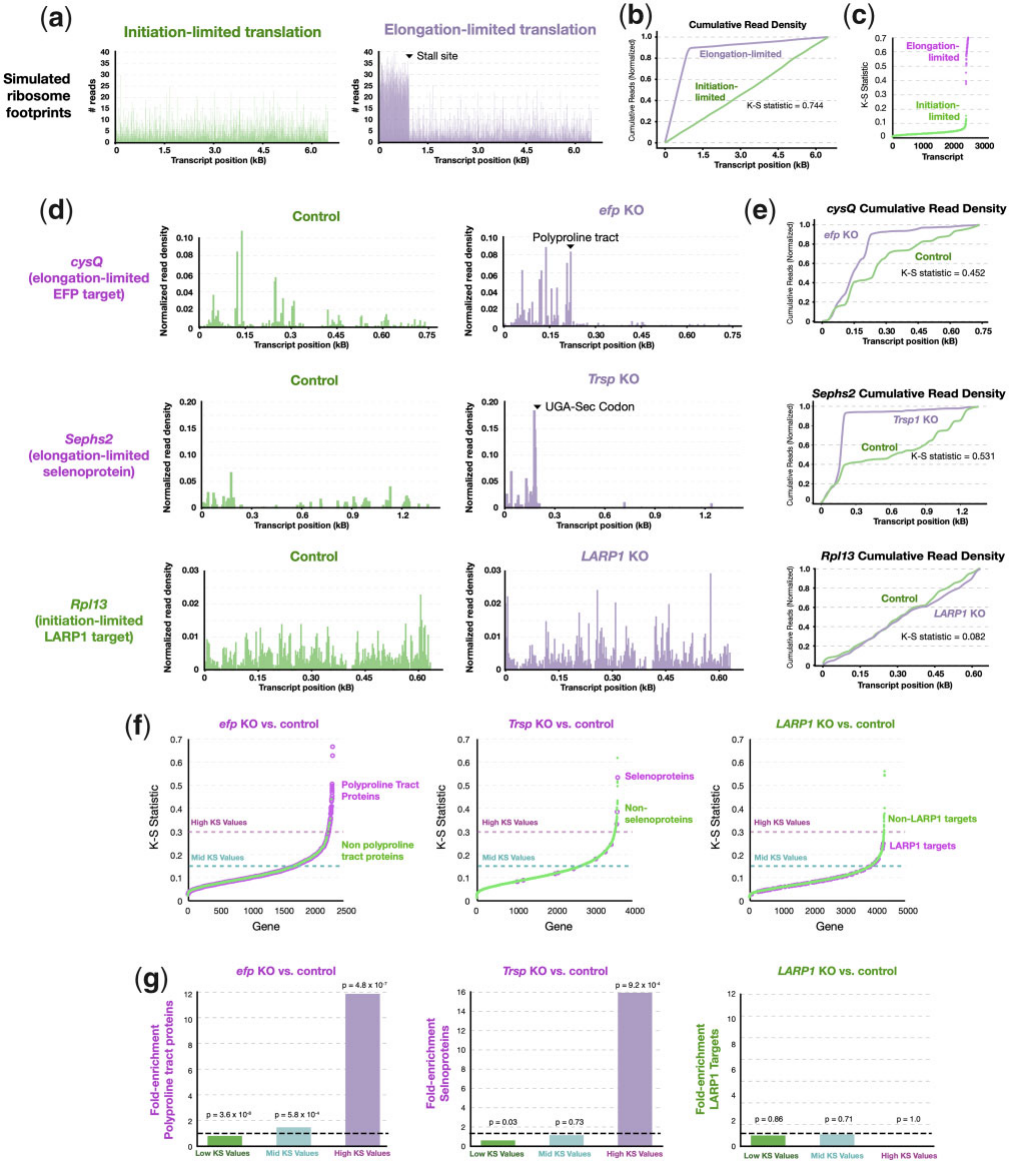
Elongation limitation leads to a quantifiable change in the distribution of ribosome footprints. a) Simulated ribosome footprinting profiles of initiation-limited and elongation-limited transcript using the inhomogenous ℓ-TASEP model (Erdmann-Pham *et al.* 2020). b) Cumulative distribution plots showing a large difference in the distribution of ribosome footprints (high K–S scores) in a simulated elongation-limited vs initiation-limited transcript. c) Plot showing high K–S values derived from comparisons (as in b) between elongation-limited but not initiation-limited transcripts. d) Ribosome footprinting profiles of *cysQ* and *Sephs2* showing reduced ribosome densities (Woolstenhulme *et al.* 2015; Fradejas-Villar *et al.* 2017b) following stall sites induced in cells lacking *efp* and *Trsp*, respectively, whereas no stall is detected for the *LARP1* target (Philippe *et al.* 2020) Rpl13. e) Cumulative distribution plots of (d) showing large differences in the distribution of ribosome footprints for *cysQ and Sephs2* but not *Rpl13* in mutant vs control cells. f, g) Plots showing that genes with known stall sites, *efp* targets and selenoproteins, have high K–S scores >0.3 in cells lacking *efp* and *Trsp*, respectively. *LARP1* targets, which have altered translation initiation, in *LARP1* KO cells do not have K–S scores >0.3.

To illustrate this phenomenon, we simulated the effect of ribosomal pauses on ribosome footprinting data from initiation- or elongation-limited genes using a biophysical model of translation (Fig. 3a). This model derives from a stochastic process of interacting particles, called the TASEP, and has been widely used for the past few years to simulate and analyze the determinants of translation speed from ribosome profiles (Zur and Tuller 2016; Dao Duc and Song 2018; Sharma *et al.* 2019; Szavits-Nossan and Ciandrini 2020). More precisely, one can distinguish under the TASEP model a traffic regime of “low density,” where the flux of ribosome is limited by translation initiation, and leads to a fairly even distribution of ribosomes across the transcript (Fig. 3a). In contrast, a “maximal current” regime is observed when the flux is limited by elongation at the region of minimal elongation rate, with a strong asymmetry in ribosome footprints accumulating upstream in this region (Fig. 3a) as predicted (Erdmann-Pham *et al.* 2020). Therefore, a slowdown of ribosome traffic within the coding region only results in a transition from initiation- to elongation-limited transition if the corresponding profiles display a discontinuous “phase transition” with asymmetry appearing at the pausing site. For initiation-limited transcripts, even if elongation rates are altered, they will not substantially affect overall translation rates unless they become rate-limiting and vice versa.

To detect differences in the processes limiting translation (e.g. initiation/elongation) between 2 ribosome footprinting profiles, we introduced using the K–S statistic (Fig. 3b), which measures the maximal difference in the cumulative distributions of 2 samples. We found that low and high K–S statistic values were highly predictive of whether a simulated profile was in the low density or maximal current regimes, respectively (Fig. 3c and Supplementary Fig. 1), with all initiation-limited or elongation-limited transcripts having a K-S statistic value lower than or greater than 0.3, respectively (Fig. 3c).

To validate the utility of this approach, we applied our method to published ribosome footprinting datasets of mutant cells lacking key elongation or translation initiation factors. We analyzed datasets where gene expression was affected by changes in translation elongation: polyproline tract-containing proteins in bacteria lacking *efp*, a factor required for the efficient incorporation of prolines within polyproline stretches (Doerfel *et al.* 2013; Ude *et al.* 2013), and selenoproteins in mouse liver lacking the selenocysteine tRNA gene *Trsp* (Fradejas-Villar *et al.* 2017). We also analyzed data from cells lacking *LARP1* (Philippe *et al.* 2020), which acts to repress the translation initiation of ribosomal proteins when mTOR is inhibited. Consistent with published data, the elongation-limited EFP target *cysQ* and selenoprotein gene *Sephs2* had reduced ribosome occupancy downstream of their respective stall sites in mutant cells (Fig. 3d). In contrast, ribosomes were similarly distributed across the length of the initiation-limited *LARP1* target *Rpl13* (Fig. 3d). Consequently, the K–S scores for *cysQ* and *Sephs2* were substantially higher (0.452 and 0.534, respectively, Fig. 3e) than *Rpl13* (0.082, Fig. 3e). We found that among genes with high K–S statistic scores (K–S statistic > 0.3), *EFP* targets and selenocysteine-containing proteins were strongly enriched, ∼12-16-fold, whereas *LARP1* targets were not enriched (Fig. 2, c and d). Thus, the K–S statistic can be used to identify genes whose overall translation is altered due to ribosome pausing.

**Fig. 4.**
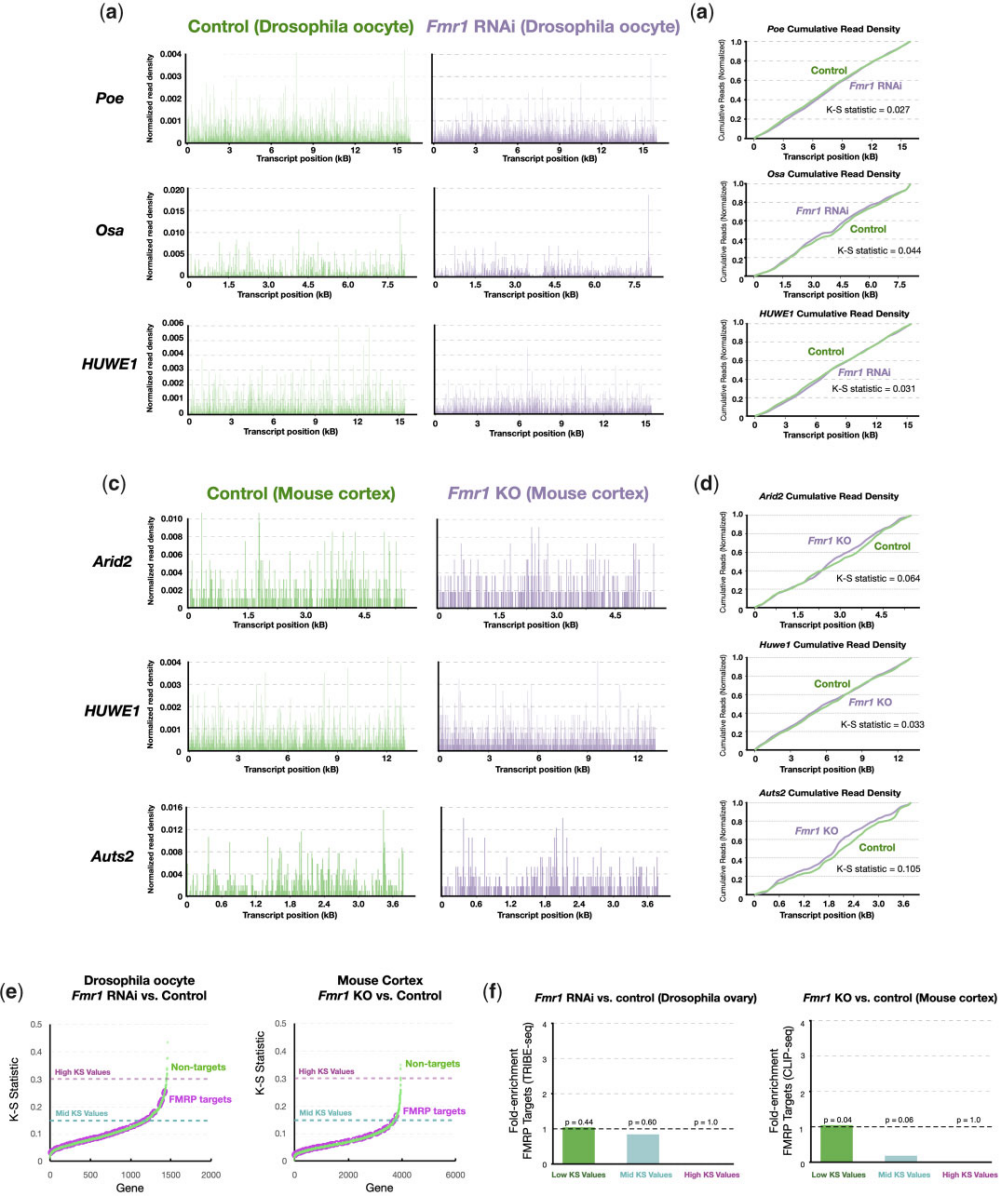
The translation of FMRP targets is limited by initiation rather than elongation rates. a) Ribosome footprints of FMRP targets *Poe*, *osa*, and *HUWE1* in control and *Fmr1* RNAi oocytes. b) Cumulative distribution plots showing nearly identical distributions of ribosome footprints for FMRP targets in wild-type and *Fmr1* RNAi oocytes. c) Ribosome footprints of mouse FMRP targets *Arid2*, *HUWE1*, and *Auts2*. f) Cumulative distribution plot showing nearly identical distributions of ribosome footprints of *Arid2*, *HUWE1*, and *Auts2* in wild-type vs FMRP-deficient tissue. e, f) Plots showing that all mouse FMRP targets as analyzed as in (d) have low K–S scores <0.3.

We performed similar tests to determine whether *Drosophila* and mouse FMRP targets are limited by translation initiation or elongation. We found that ribosome footprints along *Drosophila* targets *Poe*, *osa*, and *HUWE1* and mouse FMRP targets *Arid2*, *HUWE1*, and *Auts2* were nearly identical in their overall distributions in wild-type vs FMRP-deficient animals, with K–S statistic values varying from 0.027 to 0.105 (Fig. 4, a–d). This trend was true for other FMRP targets. Among genes with high K–S statistic values >0.3, none of the FMRP TRIBE-seq and CLIP-seq targets analyzed passed this threshold (0/111 for *Drosophila* targets and 0/103 for mouse targets) (Fig. 4, e and f). Together, our analyses do not support a major role for FMRP in controlling the translation elongation of its targets.

### ASD/ID genes encode exceptionally large proteins whose requirement for FMRP is evolutionarily conserved

**Fig. 5.**
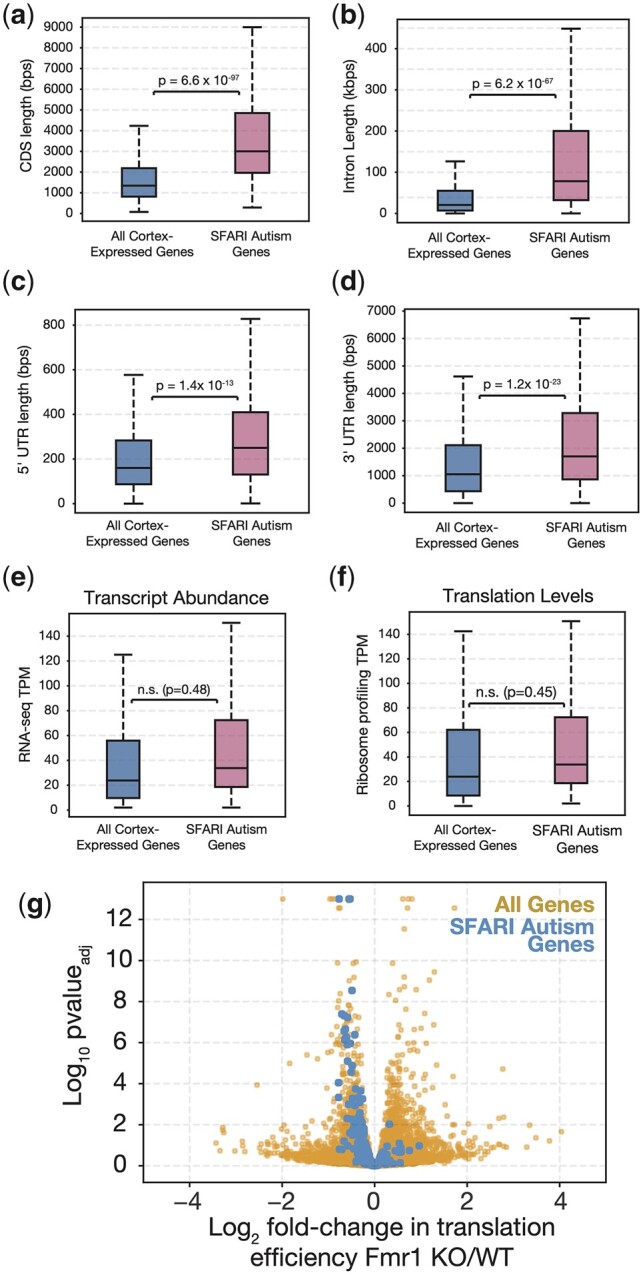
SFARI autism genes have longer open-reading frames, UTRs, and introns than average. Box plots of length distributions showing increased a) coding sequences lengths, b) 5′ UTR lengths, c) intron lengths (summed across all introns), and d) 3′ UTR lengths specifically for SFARI Class I and Class II ASD genes (magenta) as compared to all genes expressed in the juvenile mouse cortex (blue). No significant differences were detected between the distributions of transcript levels (e) or translation levels (f) specifically for ASD-relevant genes vs all cortex-expressed genes as detected by mRNA sequencing and ribosome footprinting, respectively. g) Volcano plot showing a concordant downregulation in the translation efficiency of SFARI Class I and Class II autism genes as compared to all neuronally expressed genes.

FMRP is known to associate with mRNAs from many genes associated with ASD and ID (Darnell *et al.* 2011; Sawicka *et al.* 2019) and our data indicate a specific function of FMRP RNPs in the translational activation of large proteins. To investigate whether ASD/ID genes, which are known to be exceptionally long (King *et al.* 2013), also encode proteins larger than average, we compared them with the general population of neuronally expressed genes in the juvenile mouse cortex (Das Sharma *et al.* 2019). We classified high confidence and strong candidate genes implicated in ASD—SFARI Class I, Class II, and syndromic autism genes (Abrahams *et al.* 2013; Pereanu *et al.* 2018)—as “ASD-relevant genes,” and found that they encode proteins averaging 1,234 amino acids, which is more than twice as large as the average neuronally expressed protein, 589 amino acids (Fig. 5a). There was an even greater average difference in the size of these genes (exonic and intronic sequences), 83 vs 23 kb, consistent with prior studies (King *et al.* 2013; Zhao *et al.* 2018). The greater gene size was largely because ASD-relevant genes contain larger introns totaling 78.3 kb on average vs 20.6 kb on average for neuronally expressed genes (Fig. 5b). ASD-relevant genes were only slightly larger than the neuronal average with respect to 5′ UTR length (250 vs 160 bp, Fig. 5c) and 3′ UTR length (1,703 vs 1,053 bp, Fig. 5d). ASD-relevant genes were not significantly different from other neuronal genes in mRNA levels (Fig. 5e) or translation levels (Fig. 5f). Not surprisingly given their large average CDS length, ASD-relevant genes as a class were translationally downregulated in the FMRP KO cortex (Fig. 5g). FMRP target ASD-relevant genes encode proteins even larger than the average for ASD-relevant genes generally (2,035 aa vs 1,234 aa).

### No evidence of a role for putative binding motifs in conferring FMRP target specificity

We next tested whether FMRP preferentially promotes the translation of ASD-relevant genes primarily because of their tendency to encode large proteins. If FMRP affects the translation of transcripts from ASD-relevant genes preferentially, then transcripts from these genes would be more likely to be downregulated in the *Fmr1* KO cortex as compared to transcripts of similar size from non-ASD-relevant genes. Among the 200 most significantly downregulated genes, we found that ASD-relevant genes encoding long proteins >1,800 amino acids were more enriched than ASD-relevant genes irrespective of protein size, as well as genes generally encoding large proteins (*P*-value = 0.005 and 0.02, respectively, Chi-squared test, Supplementary Fig. 2a). These data are potentially consistent with FMRP acting preferentially on a subset of large ASD-relevant target mRNAs. However, this result could also be obtained if FMRP acts nonspecifically on mRNAs encoding large proteins, if ASD-relevant genes are more likely than other genes to be expressed in cells in the cortex such as neurons that utilize FMRP-dependent translation.

Previous studies have identified short 3–4 nucleotide motifs in FMRP-bound mRNAs that are slightly enriched in FMRP-bound mRNAs and which have been proposed to contribute to FMRP binding (Ascano *et al.* 2012; Anderson *et al.* 2016). To test for a role for these motifs, we compared the frequency of putative FMRP-binding motifs WGGA, ACUK, GAC, and UAY (Anderson *et al.* 2016) in downregulated genes in the *FMR1* KO cortex as compared to nondownregulated genes. While the number of putative FMRP-binding sites was higher in downregulated genes, this was also true for any random 4-mer sequence (Supplementary Fig. 2b). When controlling for transcript length, none of the putative FMRP-binding motifs were enriched in translationally downregulated transcripts as compared to size-matched unaffected transcripts (Supplementary Fig. 2c). Thus, we find no evidence that FMRP acts preferentially on transcripts containing a high density of the putative 3–4 nucleotide-binding motifs. Our data do not exclude a model in which FMRP binds long transcripts via motifs that are present in higher frequency in targets simply because they are longer.

### Underproduction rather than overproduction of FMRP ASD target genes may lead to clinical phenotypes

The relatively modest decreases in protein production that result from the loss of FMRP function might be particularly significant in the case of “dosage-sensitive” genes that cause defects when present in only one instead of 2 genomic copies. Dosage-sensitive genes relevant for the FXS phenotype, based on the FMRP activator model, would be “haploinsufficient,” i.e. loss of either the maternal or paternal copy would lead to clinical defects. In contrast, under the repressor model relevant FMRP targets would be “triplosensitive,” where gene duplications leading to the overexpression of FMRP targets result in clinical phenotypes. We identified 61 genes that are translationally downregulated in the *Fmr1* KO mouse cortex and that score as SFARI Class I, Class II, or syndromic ASD-relevant genes (Table 1), which we considered to be potentially relevant. From this group, haploinsufficiency and triplosensitivity scores were available in the Clinical Genome Resource (NIH) database from clinical data for 32 FMRP targets (Rehm *et al.* 2015). Consistent with the activator model, we found that 22 of the 32 downregulated ASD-relevant genes were classified as haploinsufficient at the levels of “emerging evidence” and “sufficient evidence” (score of 2 and 3 respectively, Table 1). In contrast, and in contradiction to the FMRP repressor model, none of these genes (0/32) had emerging or sufficient evidence of triplosensitivity. These data indicate that reduced expression, but not overexpression, of FMRP ASD targets lead to observable clinical phenotypes and further support our model that FMRP acts primarily as an activator and not a repressor of gene expression.

**Table 1.** SFARI Class I and Class II ASD-relevant genes are translationally reduced in the *Fmr1* KO mouse cortex.

SFARI autism gene	Fold-change TE Fmr1 KO/WT	*P*-value_adj_	# amino acids	Darnell *et al.* (2011) **CLIP target**	ClinGen haplo-insufficiency score	ClinGen triplo-sensitivity score
PRR12	0.51	0.0E+00	2,035	✓	n/a	n/a
KMT2A	0.58	9.0E−05	3,966	✓	3	0
HIVEP3	0.58	4.7E−04	2,348	✓	1	0
SHANK1	0.59	0.0E+00	2,167	✓	1	0
SETD1B	0.59	1.4E−03	1,985		n/a	n/a
KMT2C	0.61	4.0E−08	4,904	✓	3	0
SPEN	0.63	2.7E−07	3,620	✓	1	0
MED13L	0.63	4.8E−08	2,207	✓	3	1
TCF20	0.64	7.1E−07	1,987	✓	3	0
SCAF4	0.64	6.1E−04	1,209		n/a	n/a
AHDC1	0.65	2.2E−07	1,594	✓	3	0
SRCAP	0.65	6.2E−07	3,271		1	0
CIC	0.66	5.4E−07	1,604	✓	2	0
KDM3B	0.66	1.3E−06	1,762		n/a	n/a
CNOT3	0.66	5.0E−03	751		0	0
MAP1A	0.67	0.0E+00	2,776	✓	n/a	n/a
SHANK2	0.67	5.9E−08	1,472	✓	2	0
RIMS1	0.67	9.7E−07	1,190		1	0
AUTS2	0.67	8.0E−06	1,261	✓	3	0
RERE	0.68	1.0E−03	1,558	✓	n/a	n/a
SYN1	0.68	1.2E−06	670	✓	2	0
HECTD4	0.68	0.0E+00	4,418		n/a	n/a
LRP1	0.69	0.0E+00	4,545	✓	n/a	n/a
CAMK2A	0.69	3.0E−04	478	✓	1	0
CREBBP	0.69	1.1E−06	2,441	✓	3	0
NOVA2	0.70	2.6E−03	492		n/a	n/a
CHD8	0.70	2.7E−05	2,582	✓	3	0
SHANK3	0.71	5.4E−03	1,805	✓	3	0
ARID2	0.71	7.3E−04	1,828	✓	3	0
ASH1L	0.71	2.9E−09	2,958	✓	3	0
IQSEC2	0.71	1.3E−05	1,479	✓	3	0
ZC3H4	0.72	3.6E−03	1,255	✓	n/a	n/a
SON	0.72	5.2E−04	2,444	✓	3	0
GRIK5	0.72	5.6E−03	979	✓	n/a	n/a
CACNA1A	0.72	1.6E−05	2,327	✓	3	0
KIF5C	0.73	1.4E−10	956	✓	n/a	n/a
HUWE1	0.73	6.6E−10	4,378	✓	0	0
ZMIZ1	0.74	1.5E−03	1,066	✓	n/a	n/a
AKAP9	0.74	1.2E−03	3,779	✓	n/a	n/a
NCOR1	0.74	4.1E−07	2,454	✓	n/a	n/a
ARID1B	0.75	1.8E−04	2,244	✓	3	0
AGO2	0.76	1.2E−03	860		n/a	n/a
MYT1L	0.77	6.6E−03	1,185	✓	3	0
FOXG1	0.78	8.6E−03	481		3	0
MECP2	0.78	9.5E−03	484		n/a	n/a
WDFY3	0.78	6.5E−06	3,508	✓	n/a	n/a
VAMP2	0.78	6.7E−05	116	✓	n/a	n/a
NSD1	0.79	4.3E−03	2,691	✓	3	0
CHD3	0.79	4.8E−04	2,055	✓	n/a	n/a
EP400	0.80	8.2E−04	2,999	✓	n/a	n/a
INTS1	0.80	5.2E−03	2,222	✓	n/a	n/a
PHF3	0.80	2.7E−03	2,025		1	0
YWHAG	0.80	5.8E−04	247	✓	n/a	n/a
SLC12A5	0.81	5.0E−03	1,138	✓	n/a	n/a
CTNND2	0.81	2.3E−04	1,221	✓	2	0
SATB1	0.82	7.4E−03	764		n/a	n/a
TRRAP	0.82	2.0E−04	3,847	✓	1	0
NFIX	0.82	5.5E−04	433	✓	n/a	n/a
SYNE1	0.82	4.1E−03	1,431	✓	n/a	n/a
TANC2	0.83	7.6E−03	1,994	✓	n/a	n/a
ATP2B2	0.83	5.5E−04	1,198	✓	n/a	n/a
ILF2	1.25	9.5E−03	390		n/a	n/a

Table showing SFARI Class I and Class II ASD−relevant genes that are significantly altered in their translation efficiency in the Fmr1 KO mouse brain. Ninety-eight percentage (61/62) of altered ASD-relevant genes are translationally reduced. ClinGen Dosage scores (Rehm *et al.* 2015) (3 = sufficient evidence, 2 = emerging evidence, 1 = little evidence, 0 = no evidence) show that many of these ASD-relevant genes have strong or emerging evidence of human syndromes associated with gene losses (haploinsufficiency) but not gene duplications (triplosensitivity). n/a, not available.

FMRP target ASD-relevant genes were predicted to be translationally downregulated to 51–83% of control levels based on reduced ribosome footprints. On average, FMRP target ASD-relevant genes were translationally reduced to similar levels as compared to generic downregulated genes (72% vs 70%, respectively). FMRP probably acts by directly binding to most of these mRNAs, since 48 of 61 (79%) were cross-linked in FMRP CLIP experiments (Darnell *et al.* 2011). Thus, at least 22 dosage-sensitive ASD-relevant genes may be translationally downregulated in the *Fmr1* KO cortex, likely through the loss of FMRP direct binding, each potentially contributing to defects despite relatively small individual reductions.

## Discussion

### FMRP has a conserved function to promote the translation of a subset of large proteins

Oocytes and neurons use common mechanisms of RNA transport and translational regulation to control protein production in both time and space. In oocytes, FMRP is required for the constitutive translation of large proteins (Greenblatt and Spradling 2018), and our analyses here suggest a similar role for FMRP in the mammalian brain. Our side-by-side comparison of ribosome profiling data shows that the effect of FMRP loss on translation in *Drosophila* oocytes and mouse cortex is extremely similar despite the fact that different tissues and organisms are involved. In both cases, the targets are more than 3 times longer than average-sized mRNAs and encode correspondingly larger proteins, including many orthologs. In both cases, the normal stimulatory effects of FMRP are estimated to be 2-fold or less, independent of the length of the mRNA and their predicted FMRP-binding site content. Moreover, based on our analyses, these similar effects do not appear to be to changes in rates of ribosome elongation or stalling, but represent differences in translation initiation and protein output. Consistent with our findings here, western analyses documented the expected reductions in 2 FMRP target proteins in oocytes, the 2,053-amino-acid snRNA 3′ processing factor IntS1 and the 5,322-amino-acid E3 ubiquitin ligase Poe/UBR4 (Greenblatt and Spradling 2018), while the 3,719-amino-acid protein kinase A regulator rugose/NBEA is reduced in *Fmr1* mutant *Drosophila* mushroom body (Sears *et al.* 2019), as well as mouse cortex FMRP targets in this study which include Auts2, Med13L, HUWE1, and Arid2 (Fig. 2, a–d).

These findings are consistent with the important and ancient role FMRP plays in neurons, oocytes, and spermatocytes from diverse animals ranging from *Drosophila* to humans (Hagerman *et al.* 2017; Drozd *et al.* 2018). Mammalian and *Drosophila* FMRP contain the same protein domains and the expression of human FMRP rescues neural defects in *Drosophila Fmr1* mutants (Coffee *et al.* 2010). FMRP had long been considered primarily a general translational repressor (Laggerbauer *et al.* 2001; Li *et al.* 2001; Darnell *et al.* 2011); however, bulk translation in the brain has been observed to be unaffected in the brains of FXS patients (Schmidt *et al.* 2020). Our data indicate that FMRP likely acts directly on its targets to increase translation. FMRP was found to bind to Poe mRNA in neurons and glia *in vivo* as well as in cultured Schneider 2 cells (McMahon *et al.* 2016) as well as to the mRNA of mouse ortholog Ubr4 in hippocampal neurons (Sawicka *et al.* 2019), and FMRP direct targets are concordantly reduced in translation in both *Drosophila* ovaries (Fig. 1c) as well as the mouse cortex (Fig. 1c). These data are corroborated by previous Ribo-tag studies of Fmr1 KO hippocampal cells, which similarly showed that 11/12 differentially expressed FMRP targets are reduced rather than increased in expression (Ceolin *et al.* 2017). Our findings here are similarly consistent with observations that FMRP CLIP targets have concordantly reduced ribosome footprints (Das Sharma *et al.* 2019), that FMRP preferentially binds to long transcripts (Sawicka *et al.* 2019; Li *et al.* 2020), and that the protein levels of large proteins are reduced rather than increased in the *Fmr1* KO hippocampal proteome (Seo *et al.* 2022). Our data are also consistent with other prior reports that FMRP activates the translation of its targets (Bechara *et al.* 2009; Tabet *et al.* 2016). Indeed, none of the mRNAs with increased translation in *Fmr1*-null animals were among the top 200 CLIP targets (Darnell *et al.* 2011; Das Sharma *et al.* 2019). The increased translation of some genes in *Fmr1* mutant animals is likely due to secondary, indirect effects of FMRP loss. FMRP targets include transcription repressors, and E3 ubiquitin ligases that stimulate protein degradation via the proteasome, while some proteins increase in translation due to the dysregulation of mTOR signaling (Sharma *et al.* 2010; Das Sharma *et al.* 2019). These data help explain the failure of the mTOR inhibitor rapamycin to reverse behavior deficits in *Fmr1*-null mice (Saré *et al.* 2017), since treating the increased translation levels of secondary FMRP targets would not rescue the primary translational defect in these animals—the underproduction of dozens of ASD/ID-associated large proteins. These data are also consistent with observations that underproduction, but not overproduction, of FMRP ASD targets leads to clinical phenotypes (Table 1).

### FMRP is used to support translation in neurons and other cells that depend on stored mRNAs

Large cells such as neurons and oocytes rely heavily on the transport and storage of stable, untranslated mRNAs. For example, a large fraction of oocyte mRNAs are translationally repressed for later use during early embryonic development, including CNS development (Kronja *et al.* 2014; Greenblatt *et al.* 2019). Neurons transport translationally regulated synaptic mRNAs up to a meter or more down long axonal projections. Such transport requires cells to partition mRNAs into repressed or actively translated states through the use of RNP particles, including P bodies and neuronal particles, that are structurally related to stress granules. These complexes often associate binding partners together through phase separation, which is sensitive to protein concentration (Molliex *et al.* 2015). Thus, there are periods when mRNAs undergoing transport from the cell body along an axon, or while stored in the vicinity of a synapse, must remain functional while in an inactive state. The rate of growth of *Drosophila* oocytes varies strongly in response to available nutrition, and both oocytes and neurons grow at very different rates at different stages of development. P bodies and stress granules can be seen to form and disperse within minutes when growth conditions vary sharply (Shimada *et al.* 2011; Wheeler *et al.* 2016). FMRP function is particularly required under conditions where development is slowed, for example after mature oocytes enter quiescence (Greenblatt and Spradling 2018). In such cells, long mRNAs are inefficiently translated as compared to shorter mRNAs (Greenblatt and Spradling 2018). *Fmr1* may have evolved during animal evolution to support the production of large proteins which play critical roles in the reproductive and nervous systems. This model is consistent with FMRP′s preferential binding to mRNA CDS regions (Darnell *et al.* 2011; Maurin *et al.* 2018). Proteins do not have to bind to the 5′ UTR or mRNA cap to affect translation initiation—for example the repressive cap-binding complex 4EHP-GYF2 is recruited to the 3′ UTR of AU-rich element-containing mRNAs through tristetraprolin to compete with eIF4E-dependent translation initiation (Fu *et al.* 2016). Another example is poly(A)-binding protein, which stimulates translation initiation through the binding of the poly(A) tail of mRNAs (Sachs and Davis 1989).

Previous observations showed that genes expressed during neural development on average encode larger proteins than in many other tissues (Gabel *et al.* 2015). Proteins regulated by FMRP are more than 3 times larger still and include many genes associated with autism and ID syndromes. There is currently no well-understood functional explanation for what exceptionally large protein size contributes to higher neural functions. Neural development likely involves complex tasks such as integrating sensory inputs and outputs in ways that are vital to survival. It may be advantageous to place multiple protein domains along a single polypeptide chain to ensure they are present at equimolar levels to help guarantee the proper stoichiometries of protein domains that function together in a complex process. Another possibility is that large neural proteins function as scaffolds for exceptionally large and specific protein complexes that play critical functions essential for neural processing. Such proteins might require many critical binding sites for additional binding partners spaced substantial distances apart along the primary sequence. If processes associated with the most complex neuronal-based decision-making are especially dependent on complexes catalyzed by enormous proteins, this might explain the enrichment of the ASD/ID gene products among the largest cohorts of FMRP targets.

## Data availability

All sequencing data were obtained from public NCBI GEO and BioProject databases with the following accessions: GSE64488 (Woolstenhulme *et al.* 2015), GSE114064 (Das Sharma *et al.* 2019), GSE132703 (Philippe *et al.* 2020), GSE84112 (Fradejas-Villar *et al.* 2017), and PRJNA466150 (Greenblatt and Spradling 2018). The compiled translation efficiency data for the *Fmr1* RNAi *Drosophila* ovary and *Fmr1* KO mouse cortex analyses are available in Supplementary Table 1. A compiled table showing the effect of *Fmr1* RNAi on the translation of *Drosophila* orthologs of ASD-relevant genes is available in Supplementary Table 2. K–S statistic values computed for *Drosophila* and mouse transcripts in comparisons of control and *Fmr1*-deficient tissues are available in Supplementary Table 3. Unprocessed RNA sequencing and ribosome profiling TPM values are available in Supplementary Table 4.

Supplemental material is available at *GENETICS* online.

## Funding

This work was supported by funding through the Howard Hughes Medical Institute (Q.Y., A.C.S., and E.G.), the Simons Foundation Autism Research Initiative (K.F., A.B.H., and E.G.), and Michael Smith Health Research BC (E.G.).

## Conflicts of interest

None declared.

## Supplementary Material

iyac094_Supplementary_Table_1

iyac094_Supplementary_Table_2

iyac094_Supplementary_Table_3

iyac094_Supplementary_Table_4

iyac094_Supplementary_Table_5

iyac094_Supplemental_Figures_and_Legends

## References

[iyac094-B1] Abrahams BS , Arking DE, Campbell DB, Mefford HC, Morrow EM, Weiss LA, Menashe I, Wadkins T, Banerjee-Basu S, Packer A. SFARI Gene 2.0: a community-driven knowledgebase for the autism spectrum disorders (ASDs). Mol Autism. 2013;4(1):36. doi:10.1186/2040-2392-4-36. 24090431 PMC3851189

[iyac094-B2] Anderson BR , Chopra P, Suhl JA, Warren ST, Bassell GJ. Identification of consensus binding sites clarifies FMRP binding determinants. Nucleic Acids Res. 2016;44(14):6649–6659. doi:10.1093/nar/gkw593. 27378784 PMC5001617

[iyac094-B3] Aryal S , Klann E. Turning up translation in fragile X syndrome. Science. 2018;361(6403):648–649. doi:10.1126/science.aau6450. 30115797

[iyac094-B4] Aryal S , Longo F, Klann E. Genetic removal of p70 S6K1 corrects coding sequence length-dependent alterations in mRNA translation in fragile X syndrome mice. Proc Natl Acad Sci USA. 2021;118(18). doi:10.1073/pnas.2001681118. PMC8106352 33906942

[iyac094-B5] Ascano M , Mukherjee N, Bandaru P, Miller JB, Nusbaum JD, Corcoran DL, Langlois C, Munschauer M, Dewell S, Hafner M, et al FMRP targets distinct mRNA sequence elements to regulate protein expression. Nature. 2012;492(7429):382–386. doi:10.1038/nature11737. 23235829 PMC3528815

[iyac094-B6] Baradaran-Heravi A , Balgi AD, Zimmerman C, Choi K, Shidmoossavee FS, Tan JS, Bergeaud C, Krause A, Flibotte S, Shimizu Y, et al Novel small molecules potentiate premature termination codon readthrough by aminoglycosides. Nucleic Acids Res. 2016;44(14):6583–6598. doi:10.1093/nar/gkw638. 27407112 PMC5001621

[iyac094-B7] Bechara EG , Didiot MC, Melko M, Davidovic L, Bensaid M, Martin P, Castets M, Pognonec P, Khandjian EW, Moine H, et al A novel function for fragile X mental retardation protein in translational activation. PLoS Biol. 2009;7(1):e16. doi:10.1371/journal.pbio.1000016. 19166269 PMC2628407

[iyac094-B8] Brager DH , Akhavan AR, Johnston D. Impaired dendritic expression and plasticity of h-channels in the fmr1(-/y) mouse model of fragile X syndrome. Cell Rep. 2012;1(3):225–233. doi:10.1016/j.celrep.2012.02.002. 22662315 PMC3363364

[iyac094-B9] Bramswig NC , Caluseriu O, Lüdecke H-J, Bolduc FV, Noel NCL, Wieland T, Surowy HM, Christen H-J, Engels H, Strom TM, et al Heterozygosity for ARID2 loss-of-function mutations in individuals with a Coffin-Siris syndrome-like phenotype. Hum Genet. 2017;136(3):297–305. doi:10.1007/s00439-017-1757-z. 28124119

[iyac094-B10] Bülow P , Murphy TJ, Bassell GJ, Wenner P. Homeostatic intrinsic plasticity is functionally altered in fmr1 KO cortical neurons. Cell Rep. 2019;26(6):1378–1388.e3. doi:10.1016/j.celrep.2019.01.035. 30726724 PMC6443253

[iyac094-B11] Callan MA , Cabernard C, Heck J, Luois S, Doe CQ, Zarnescu DC. Fragile X protein controls neural stem cell proliferation in the Drosophila brain. Hum Mol Genet. 2010;19(15):3068–3079. doi:10.1093/hmg/ddq213. 20504994 PMC2901145

[iyac094-B12] Ceolin L , Bouquier N, Vitre-Boubaker J, Rialle S, Severac D, Valjent E, Perroy J, Puighermanal E. Cell type-specific mRNA dysregulation in hippocampal CA1 pyramidal neurons of the fragile X syndrome mouse model. Front Mol Neurosci. 2017;10:340. doi:10.3389/fnmol.2017.00340. 29104533 PMC5655025

[iyac094-B13] Coffee RL , Tessier CR, Woodruff EA, Broadie K. Fragile X mental retardation protein has a unique, evolutionarily conserved neuronal function not shared with FXR1P or FXR2P. Dis Models Mech. 2010;3(7–8):471–485. doi:10.1242/dmm.004598. PMC2898537 20442204

[iyac094-B14] Dao Duc K , Song YS. The impact of ribosomal interference, codon usage, and exit tunnel interactions on translation elongation rate variation. PLoS Genet. 2018;14(1):e1007166. doi:10.1371/journal.pgen.1007166. 29337993 PMC5786338

[iyac094-B15] Darnell AM , Subramaniam AR, O'Shea EK. Translational control through differential ribosome pausing during amino acid limitation in mammalian cells. Mol Cell. 2018;71(2):229–243.e11. doi:10.1016/j.molcel.2018.06.041. 30029003 PMC6516488

[iyac094-B16] Darnell JC , Van Driesche SJ, Zhang C, Hung KYS, Mele A, Fraser CE, Stone EF, Chen C, Fak JJ, Chi SW, et al FMRP stalls ribosomal translocation on mRNAs linked to synaptic function and autism. Cell. 2011;146(2):247–261. doi:10.1016/j.cell.2011.06.013. 21784246 PMC3232425

[iyac094-B17] Das Sharma S , Metz JB, Li H, Hobson BD, Hornstein N, Sulzer D, Tang G, Sims PA. Widespread alterations in translation elongation in the brain of juvenile fmr1 knockout mice. Cell Rep. 2019;26(12):3313–3322.e5. doi:10.1016/j.celrep.2019.02.086. 30893603 PMC6457272

[iyac094-B18] Dobin A , Davis CA, Schlesinger F, Drenkow J, Zaleski C, Jha S, Batut P, Chaisson M, Gingeras TR. STAR: ultrafast universal RNA-seq aligner. Bioinformatics. 2013;29(1):15–21. doi:10.1093/bioinformatics/bts635. 23104886 PMC3530905

[iyac094-B19] Doerfel LK , Wohlgemuth I, Kothe C, Peske F, Urlaub H, Rodnina MV. EF-P is essential for rapid synthesis of proteins containing consecutive proline residues. Science. 2013;339(6115):85–88. doi:10.1126/science.1229017. 23239624

[iyac094-B20] Drozd M , Bardoni B, Capovilla M. Modeling fragile X syndrome in Drosophila. Front Mol Neurosci. 2018;11:124. doi:10.3389/fnmol.2018.00124. 29713264 PMC5911982

[iyac094-B21] Dunn JG , Weissman JS. Plastid: nucleotide-resolution analysis of next-generation sequencing and genomics data. BMC Genomics. 2016;17(1):958. doi:10.1186/s12864-016-3278-x. 27875984 PMC5120557

[iyac094-B22] Erdmann-Pham DD , Dao Duc K, Song YS. The key parameters that govern translation efficiency. Cell Syst. 2020;10(2):183–192.e6. doi:10.1016/j.cels.2019.12.003. 31954660 PMC7047610

[iyac094-B23] Erdmann-Pham DD , Son W, Dao Duc K, Song YS. EGGTART: a tool to visualize the dynamics of biophysical transport under the inhomogeneous l-TASEP. Biophys J. 2021;120(8):1309–1313. doi:10.1016/j.bpj.2021.02.004. 33582139 PMC8105713

[iyac094-B24] Fradejas-Villar N , Seeher S, Anderson CB, Doengi M, Carlson BA, Hatfield DL, Schweizer U, Howard MT. The RNA-binding protein Secisbp2 differentially modulates UGA codon reassignment and RNA decay. Nucleic Acids Res. 2017;45(7):4094–4107. doi:10.1093/nar/gkw1255. 27956496 PMC5397149

[iyac094-B25] Fu R , Olsen MT, Webb K, Bennett EJ, Lykke-Andersen J. Recruitment of the 4EHP-GYF2 cap-binding complex to tetraproline motifs of tristetraprolin promotes repression and degradation of mRNAs with AU-rich elements. RNA. 2016;22(3):373–382. doi:10.1261/rna.054833.115. 26763119 PMC4748815

[iyac094-B26] Gabel HW , Kinde B, Stroud H, Gilbert CS, Harmin DA, Kastan NR, Hemberg M, Ebert DH, Greenberg ME. Disruption of DNA-methylation-dependent long gene repression in Rett syndrome. Nature. 2015;522(7554):89–93. doi:10.1038/nature14319. 25762136 PMC4480648

[iyac094-B27] Gao Z , Lee P, Stafford JM, von Schimmelmann M, Schaefer A, Reinberg D. An AUTS2-Polycomb complex activates gene expression in the CNS. Nature. 2014;516(7531):349–354. doi:10.1038/nature13921. 25519132 PMC4323097

[iyac094-B28] Goering R , Hudish LI, Guzman BB, Raj N, Bassell GJ, Russ HA, Dominguez D, Taliaferro JM. FMRP promotes RNA localization to neuronal projections through interactions between its RGG domain and G-quadruplex RNA sequences. eLife. 2020;9. doi:10.7554/eLife.52621. PMC7279889 32510328

[iyac094-B29] Greenblatt EJ , Obniski R, Mical C, Spradling AC. Prolonged ovarian storage of mature Drosophila oocytes dramatically increases meiotic spindle instability. eLife. 2019;8. doi:10.7554/eLife.49455. PMC6905857 31755866

[iyac094-B30] Greenblatt EJ , Spradling AC. Fragile X mental retardation 1 gene enhances the translation of large autism-related proteins. Science. 2018;361(6403):709–712. doi:10.1126/science.aas9963. 30115809 PMC6905618

[iyac094-B31] Grossman AW , Elisseou NM, McKinney BC, Greenough WT. Hippocampal pyramidal cells in adult Fmr1 knockout mice exhibit an immature-appearing profile of dendritic spines. Brain Res. 2006;1084(1):158–164. doi:10.1016/j.brainres.2006.02.044. 16574084

[iyac094-B32] Hagerman RJ , Berry-Kravis E, Hazlett HC, Bailey DB, Moine H, Kooy RF, Tassone F, Gantois I, Sonenberg N, Mandel JL, et al Fragile X syndrome. Nat Rev Dis Primers. 2017;3:17065. doi:10.1038/nrdp.2017.65. 28960184

[iyac094-B33] Hays SA , Huber KM, Gibson JR. Altered neocortical rhythmic activity states in *Fmr1* KO mice are due to enhanced mGluR5 signaling and involve changes in excitatory circuitry. J Neurosci. 2011;31(40):14223–14234. doi:10.1523/JNEUROSCI.3157-11.2011. 21976507 PMC3207280

[iyac094-B34] Hsu PJ , Shi H, Zhu AC, Lu Z, Miller N, Edens BM, Ma YC, He C. The RNA-binding protein FMRP facilitates the nuclear export of N6-methyladenosine-containing mRNAs. J Biol Chem. 2019;294(52):19889–19895. doi:10.1074/jbc.AC119.010078. 31753916 PMC6937581

[iyac094-B35] Ingolia NT , Lareau LF, Weissman JS. Ribosome profiling of mouse embryonic stem cells reveals the complexity and dynamics of mammalian proteomes. Cell. 2011;147(4):789–802. doi:10.1016/j.cell.2011.10.002. 22056041 PMC3225288

[iyac094-B36] Kao D-I , Aldridge GM, Weiler IJ, Greenough WT. Altered mRNA transport, docking, and protein translation in neurons lacking fragile X mental retardation protein. Proc Natl Acad Sci U S A. 2010;107(35):15601–15606. doi:10.1073/pnas.1010564107. 20713728 PMC2932564

[iyac094-B37] Khong A , Matheny T, Jain S, Mitchell SF, Wheeler JR, Parker R. The stress granule transcriptome reveals principles of mRNA accumulation in stress granules. Mol Cell. 2017;68(4):808–820.e5. doi:10.1016/j.molcel.2017.10.015. 29129640 PMC5728175

[iyac094-B38] King IF , Yandava CN, Mabb AM, Hsiao JS, Huang H-S, Pearson BL, Calabrese JM, Starmer J, Parker JS, Magnuson T, et al Topoisomerases facilitate transcription of long genes linked to autism. Nature. 2013;501(7465):58–62. doi:10.1038/nature12504. 23995680 PMC3767287

[iyac094-B39] Kronja I , Yuan B, Eichhorn SW, Dzeyk K, Krijgsveld J, Bartel DP, Orr-Weaver TL. Widespread changes in the posttranscriptional landscape at the Drosophila oocyte-to-embryo transition. Cell Rep. 2014;7(5):1495–1508. doi:10.1016/j.celrep.2014.05.002. 24882012 PMC4143395

[iyac094-B40] Laggerbauer B , Ostareck D, Keidel EM, Ostareck-Lederer A, Fischer U. Evidence that fragile X mental retardation protein is a negative regulator of translation. Hum Mol Genet. 2001;10(4):329–338. doi:10.1093/hmg/10.4.329. 11157796

[iyac094-B41] Lauria F , Tebaldi T, Bernabò P, Groen EJN, Gillingwater TH, Viero G. riboWaltz: optimization of ribosome P-site positioning in ribosome profiling data. PLoS Comput Biol. 2018;14(8):e1006169. doi:10.1371/journal.pcbi.1006169. 30102689 PMC6112680

[iyac094-B42] Li M , Shin J, Risgaard RD, Parries MJ, Wang J, Chasman D, Liu S, Roy S, Bhattacharyya A, Zhao X. Identification of FMR1-regulated molecular networks in human neurodevelopment. Genome Res. 2020;30(3):361–374. doi:10.1101/gr.251405.119. 32179589 PMC7111522

[iyac094-B43] Li Z , Zhang Y, Ku L, Wilkinson KD, Warren ST, Feng Y. The fragile X mental retardation protein inhibits translation via interacting with mRNA. Nucleic Acids Res. 2001;29(11):2276–2283. doi:10.1093/nar/29.11.2276. 11376146 PMC55699

[iyac094-B44] Liu B , Li Y, Stackpole EE, Novak A, Gao Y, Zhao Y, Zhao X, Richter JD. Regulatory discrimination of mRNAs by FMRP controls mouse adult neural stem cell differentiation. Proc Natl Acad Sci U S A. 2018;115(48):E11397–E11405. doi:10.1073/pnas.1809588115. 30373821 PMC6275535

[iyac094-B45] Maurin T , Lebrigand K, Castagnola S, Paquet A, Jarjat M, Popa A, Grossi M, Rage F, Bardoni B. HITS-CLIP in various brain areas reveals new targets and new modalities of RNA binding by fragile X mental retardation protein. Nucleic Acids Res. 2018;46(12):6344–6355. doi:10.1093/nar/gky267. 29668986 PMC6158598

[iyac094-B46] McMahon AC , Rahman R, Jin H, Shen JL, Fieldsend A, Luo W, Rosbash M. TRIBE: hijacking an RNA-editing enzyme to identify cell-specific targets of RNA-binding proteins. Cell. 2016;165(3):742–753. doi:10.1016/j.cell.2016.03.007. 27040499 PMC5027142

[iyac094-B47] Molliex A , Temirov J, Lee J, Coughlin M, Kanagaraj AP, Kim HJ, Mittag T, Taylor JP. Phase separation by low complexity domains promotes stress granule assembly and drives pathological fibrillization. Cell. 2015;163(1):123–133. doi:10.1016/j.cell.2015.09.015. 26406374 PMC5149108

[iyac094-B48] Oksenberg N , Ahituv N. The role of *AUTS2* in neurodevelopment and human evolution. Trends Genet. 2013;29(10):600–608. doi:10.1016/j.tig.2013.08.001. 24008202 PMC3823538

[iyac094-B49] Osterweil EK , Krueger DD, Reinhold K, Bear MF. Hypersensitivity to mGluR5 and ERK1/2 leads to excessive protein synthesis in the hippocampus of a mouse model of fragile X syndrome. J Neurosci. 2010;30(46):15616–15627. doi:10.1523/JNEUROSCI.3888-10.2010. 21084617 PMC3400430

[iyac094-B50] Pereanu W , Larsen EC, Das I, Estévez MA, Sarkar AA, Spring-Pearson S, Kollu R, Basu SN, Banerjee-Basu S. AutDB: a platform to decode the genetic architecture of autism. Nucleic Acids Res. 2018;46(D1):D1049–D1054. doi:10.1093/nar/gkx1093. 29186576 PMC5753210

[iyac094-B51] Philippe L , G van den Elzen AM, Watson MJ, Thoreen CC. Global analysis of LARP1 translation targets reveals tunable and dynamic features of 5’ TOP motifs. Proc Natl Acad Sci U S A. 2020;117(10):5319–5328. doi:10.1073/pnas.1912864117. 32094190 PMC7071917

[iyac094-B52] Prilutsky D , Kho AT, Palmer NP, Bhakar AL, Smedemark-Margulies N, Kong SW, Margulies DM, Bear MF, Kohane IS. Gene expression analysis in *Fmr1*KO mice identifies an immunological signature in brain tissue and mGluR5-related signaling in primary neuronal cultures. Mol Autism. 2015;6:66. doi:10.1186/s13229-015-0061-9. 26697163 PMC4687343

[iyac094-B53] Rehm HL , Berg JS, Brooks LD, Bustamante CD, Evans JP, Landrum MJ, Ledbetter DH, Maglott DR, Martin CL, Nussbaum RL, et al ClinGen—the clinical genome resource. N Engl J Med. 2015;372(23):2235–2242. – doi:10.1056/NEJMsr1406261. 26014595 10.1056/NEJMsr1406261 PMC4474187

[iyac094-B54] Sachs AB , Davis RW. The poly(A) binding protein is required for poly(A) shortening and 60S ribosomal subunit-dependent translation initiation. Cell. 1989;58(5):857–867. doi:10.1016/0092-8674(89)90938-0. 2673535

[iyac094-B55] Saré RM , Song A, Loutaev I, Cook A, Maita I, Lemons A, Sheeler C, Smith CB. Negative effects of chronic rapamycin treatment on behavior in a mouse model of fragile X syndrome. Front Mol Neurosci. 2017;10:452. doi:10.3389/fnmol.2017.00452. 29375310 PMC5770365

[iyac094-B56] Sawicka K , Hale CR, Park CY, Fak JJ, Gresack JE, Van Driesche SJ, Kang JJ, Darnell JC, Darnell RB. FMRP has a cell-type-specific role in CA1 pyramidal neurons to regulate autism-related transcripts and circadian memory. eLife 8. 2019;8. doi:10.7554/eLife.46919. PMC6924960 31860442

[iyac094-B57] Schmidt EK , Clavarino G, Ceppi M, Pierre P. SUnSET, a nonradioactive method to monitor protein synthesis. Nat Methods. 2009;6(4):275–277. doi:10.1038/nmeth.1314. 19305406

[iyac094-B58] Schmidt KC , Loutaev I, Quezado Z, Sheeler C, Smith CB. Regional rates of brain protein synthesis are unaltered in dexmedetomidine sedated young men with fragile X syndrome: a L-[1-11C]leucine PET study. Neurobiol Dis. 2020;143:104978. doi:10.1016/j.nbd.2020.104978. 32569795 PMC7425798

[iyac094-B59] Schuller AP , Wu CC-C, Dever TE, Buskirk AR, Green R. eIF5A functions globally in translation elongation and termination. Mol Cell. 2017;66(2):194–205.e5. doi:10.1016/j.molcel.2017.03.003. 28392174 PMC5414311

[iyac094-B60] Sears JC , Choi WJ, Broadie K. Fragile X Mental Retardation Protein positively regulates PKA anchor Rugose and PKA activity to control actin assembly in learning/memory circuitry. Neurobiol Dis. 2019;127:53–64. doi:10.1016/j.nbd.2019.02.004. 30771457 PMC6588493

[iyac094-B61] Seo SS , Louros SR, Anstey N, Gonzalez-Lozano MA, Harper CB, Verity NC, Dando O, Thomson SR, Darnell JC, Kind PC, et al Excess ribosomal protein production unbalances translation in a model of fragile X syndrome. Nat Commun. 2022;13(1):3236. doi:10.1038/s41467-022-30979-0. 35688821 PMC9187743

[iyac094-B62] Shah P , Ding Y, Niemczyk M, Kudla G, Plotkin JB. Rate-limiting steps in yeast protein translation. Cell. 2013;153(7):1589–1601. doi:10.1016/j.cell.2013.05.049. 23791185 PMC3694300

[iyac094-B63] Sharma AK , Sormanni P, Ahmed N, Ciryam P, Friedrich UA, Kramer G, O'Brien EP. A chemical kinetic basis for measuring translation initiation and elongation rates from ribosome profiling data. PLoS Comput Biol. 2019;15(5):e1007070. doi:10.1371/journal.pcbi.1007070. 31120880 PMC6559674

[iyac094-B64] Sharma A , Hoeffer CA, Takayasu Y, Miyawaki T, McBride SM, Klann E, Zukin RS. Dysregulation of mTOR signaling in fragile X syndrome. J Neurosci. 2010;30(2):694–702. doi:10.1523/JNEUROSCI.3696-09.2010. 20071534 PMC3665010

[iyac094-B65] Shimada Y , Burn KM, Niwa R, Cooley L. Reversible response of protein localization and microtubule organization to nutrient stress during Drosophila early oogenesis. Dev Biol. 2011;355(2):250–262. doi:10.1016/j.ydbio.2011.04.022. 21570389 PMC3118931

[iyac094-B66] Shu H , Donnard E, Liu B, Jung S, Wang RRichter JD. FMRP links optimal codons to mRNA stability in neurons. Proceedings of the National Academy of Sciences of the United States of America 117:30400–30411. doi:10.1073/pnas.2009161117. PMC7720238 33199649

[iyac094-B67] Sullivan SD , Welt C, Sherman S. FMR1 and the continuum of primary ovarian insufficiency. Semin Reprod Med. 2011;29(4):299–307. doi:10.1055/s-0031-1280915. 21969264

[iyac094-B68] Szavits-Nossan J , Ciandrini L. Inferring efficiency of translation initiation and elongation from ribosome profiling. Nucleic Acids Res. 2020;48(17):9478–9490. doi:10.1093/nar/gkaa678. 32821926 PMC7515720

[iyac094-B69] Tabet R , Moutin E, Becker JAJ, Heintz D, Fouillen L, Flatter E, Krężel W, Alunni V, Koebel P, Dembélé D, et al Fragile X Mental Retardation Protein (FMRP) controls diacylglycerol kinase activity in neurons. Proc Natl Acad Sci U S A. 2016;113(26):E3619–28. doi:10.1073/pnas.1522631113. 27233938 PMC4932937

[iyac094-B70] Ude S , Lassak J, Starosta AL, Kraxenberger T, Wilson DN, Jung K. Translation elongation factor EF-P alleviates ribosome stalling at polyproline stretches. Science. 2013;339(6115):82–85. doi:10.1126/science.1228985. 23239623

[iyac094-B71] Utami KH , Winata CL, Hillmer AM, Aksoy I, Long HT, Liany H, Chew EGY, Mathavan S, Tay SKH, Korzh V, et al Impaired development of neural-crest cell-derived organs and intellectual disability caused by *MED13L* haploinsufficiency. Hum Mutat. 2014;35(11):1311–1320. doi:10.1002/humu.22636. 25137640

[iyac094-B72] Vasilyev N , Polonskaia A, Darnell JC, Darnell RB, Patel DJ, Serganov A. Crystal structure reveals specific recognition of a G-quadruplex RNA by a β-turn in the RGG motif of FMRP. Proc Natl Acad Sci U S A. 2015;112(39):E5391–400. doi:10.1073/pnas.1515737112. 26374839 PMC4593078

[iyac094-B73] Wheeler JR , Matheny T, Jain S, Abrisch R, Parker R. Distinct stages in stress granule assembly and disassembly. eLife. 2016;5. doi:10.7554/eLife.18413. PMC5014549 27602576

[iyac094-B74] Wolin SL , Walter P. Ribosome pausing and stacking during translation of a eukaryotic mRNA. EMBO J. 1988;7(11):3559–3569. doi:10.1002/j.1460-2075.1988.tb03233.x. 2850168 PMC454858

[iyac094-B75] Woolstenhulme CJ , Guydosh NR, Green R, Buskirk AR. High-precision analysis of translational pausing by ribosome profiling in bacteria lacking EFP. Cell Rep. 2015;11(1):13–21. doi:10.1016/j.celrep.2015.03.014. 25843707 PMC4835038

[iyac094-B76] Zaslavsky K , Zhang W-B, McCready FP, Rodrigues DC, Deneault E, Loo C, Zhao M, Ross PJ, El Hajjar J, Romm A, et al *SHANK2* mutations associated with autism spectrum disorder cause hyperconnectivity of human neurons. Nat Neurosci. 2019;22(4):556–564. doi:10.1038/s41593-019-0365-8. 30911184 PMC6475597

[iyac094-B77] Zhang Z , Marro SG, Zhang Y, Arendt KL, Patzke C, Zhou B, Fair T, Yang N, Südhof TC, Wernig M, et al The fragile X mutation impairs homeostatic plasticity in human neurons by blocking synaptic retinoic acid signaling. Sci Transl Med. 2018;10(452). doi:10.1126/scitranslmed.aar4338. PMC6317709 30068571

[iyac094-B78] Zhao X , Heng JI-T, Guardavaccaro D, Jiang R, Pagano M, Guillemot F, Iavarone A, Lasorella A. The HECT-domain ubiquitin ligase Huwe1 controls neural differentiation and proliferation by destabilizing the N-Myc oncoprotein. Nat Cell Biol. 2008;10(6):643–653. doi:10.1038/ncb1727. 18488021 PMC2680438

[iyac094-B79] Zhao Y-T , Kwon DY, Johnson BS, Fasolino M, Lamonica JM, Kim YJ, Zhao BS, He C, Vahedi G, Kim TH, et al Long genes linked to autism spectrum disorders harbor broad enhancer-like chromatin domains. Genome Res. 2018;28(7):933–942. doi:10.1101/gr.233775.117. 29848492 10.1101/gr.233775.117 PMC6028126

[iyac094-B80] Zhong Y , Karaletsos T, Drewe P, Sreedharan VT, Kuo D, Singh K, Wendel H-G, Rätsch G. RiboDiff: detecting changes of mRNA translation efficiency from ribosome footprints. Bioinformatics. 2017;33(1):139–141. doi:10.1093/bioinformatics/btw585. 27634950 PMC5198522

[iyac094-B81] Zur H , Tuller T. Predictive biophysical modeling and understanding of the dynamics of mRNA translation and its evolution. Nucleic Acids Res. 2016;44(19):9031–9049. doi:10.1093/nar/gkw764. 27591251 PMC5100582

