# Supplementary material for: FMRP-dependent production of large dosage-sensitive proteins is highly conserved
Source: Genetics. 2022 Jun 22;221(4):iyac094. doi: 10.1093/genetics/iyac094 (PMC9339308; doi:10.1093/genetics/iyac094)
Supplement: iyac094_Supplemental_Figures_and_Legends [file iyac094_supplemental_figures_and_legends.pdf]

SUPPLEMENTAL FIGURE LEGEND

Figure S1. The K-S statistic can distinguish between initiation and elongation-limitation for transcripts with low read coverage. (A) Plot showing K-S statistic values for simulated ribosome footprints on initiation limited mRNAs using the inhomogenous I-TASEP model as a function of read density. (B,C) Plots showing K-S statistic values for simulated ribosome footprints with varying read densities which are derived from elongation-limited mRNAs with a (B) weak pause (minimal elongation rate is just rate-limiting) and (C) a strong pause (twice the strength of the weak pause).

Figure S2. No enrichment for putative FMRP binding motifs among translationally downregulated proteins in the *Fmr1* KO mouse cortex. (A) Plot showing the fold-enrichment of SFARI class I, class II and syndromic genes and genes encoding large proteins > 1,800 amino acids among the top 200 translationally downregulated genes in the *Fmr1* KO cortex. (B) Box plots showing that the raw number of putative FMRP binding sequences are similarly enriched among translationally downregulated genes in the *Fmr1* KO mouse cortex as a random 4-mer sequence. (C) Box plots showing that the density of putative FMRP binding sequences are statistically identical for downregulated genes and non-downregulated genes encoding large proteins in the *Fmr1* KO mouse cortex.

TABLE LEGENDS

Supplementary Table 1. Compiled RiboDiff output for *Fmr1* RNAi *Drosophila* oocyte and *Fmr1* KO mouse cortex analyses.

Supplementary Table 2. ClinGen haploinsufficiency scores for *Drosophila* orthologs of SFARI Class I, Class II, and syndromic autism genes translationally downregulated in

oocytes.

Supplementary Table 3. Compiled K-S statistic values for *Drosophila* and mouse *Fmr1* ribosome profiling analyses.

Supplementary Table 4. Compiled unprocessed gene expression data for *Drosophila* and mouse *Fmr1* ribosome profiling and RNA-seq experiments.

Supplementary Table 5. P-site offsets used for processing human, mouse, and *Drosophila* ribosome profiling data.

These tables are provided separately as Excel files.

Figure S1

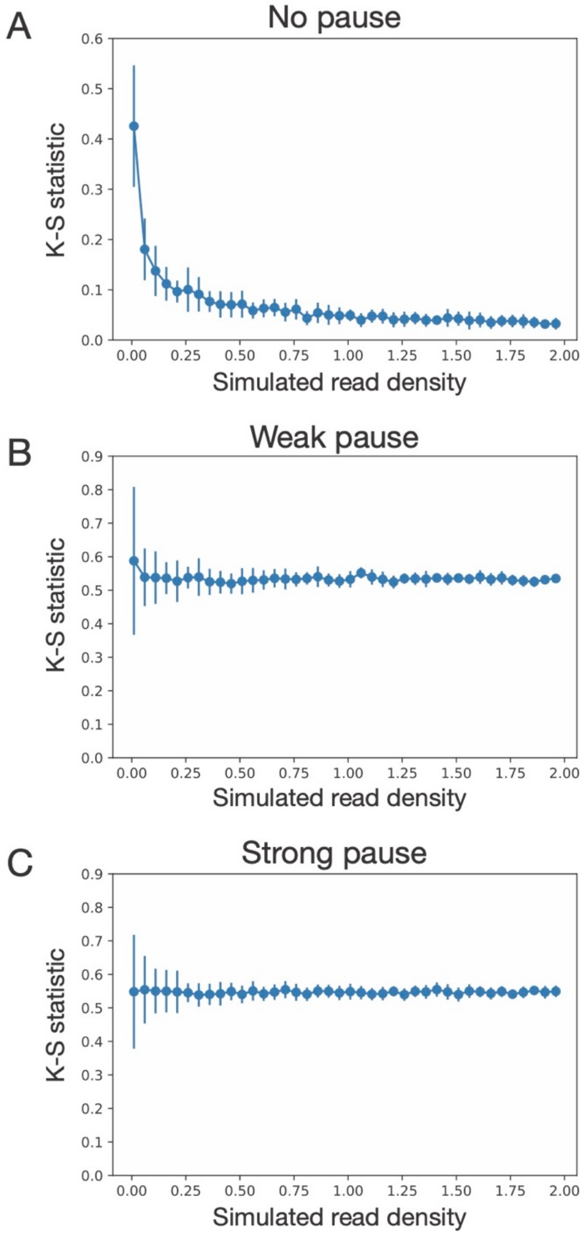
A
No pause
K-S statistic
Simulated read density
B
Weak pause
K-S statistic
Simulated read density
C
Strong pause
K-S statistic
Simulated read density

Figure S2

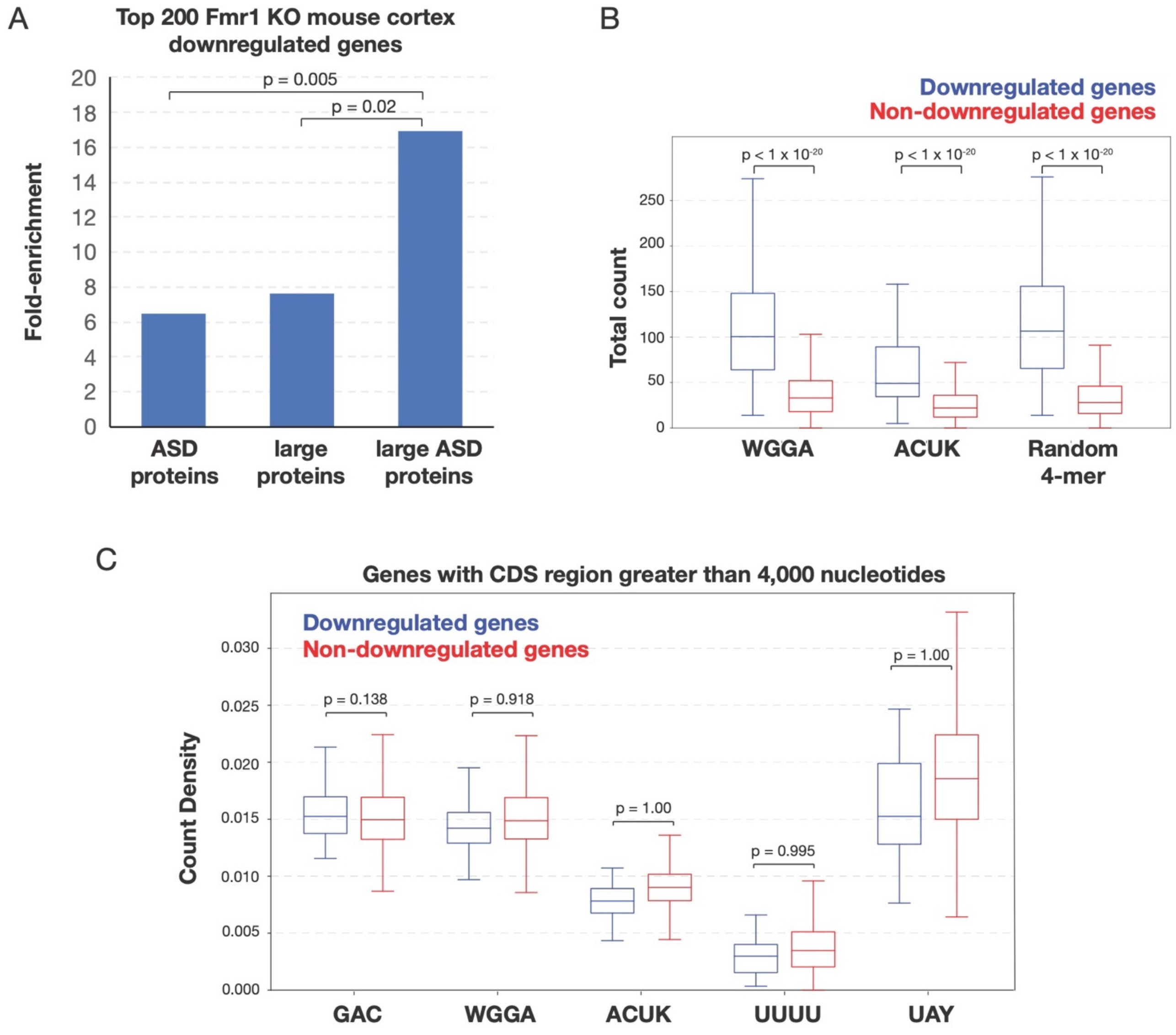
A
Top 200 Fmr1 KO mouse cortex downregulated genes
Fold-enrichment
20
18
16
14
12
10
8
6
4
2
0
p = 0.005
p = 0.02
ASD proteins
large proteins
large ASD proteins
B
Downregulated genes
Non-downregulated genes
Total count
250
200
150
100
50
0
p < 1 x 10-20
p < 1 x 10-20
p < 1 x 10-20
WGGA
ACUK
Random 4-mer
C
Genes with CDS region greater than 4,000 nucleotides
Downregulated genes
Non-downregulated genes
Count Density
0.030
0.025
0.020
0.015
0.010
0.005
0.000
p = 0.138
p = 0.918
p = 1.00
p = 0.995
p = 1.00
GAC
WGGA
ACUK
UUUU
UAY
